# A Data-Driven Reference Standard for Adverse Drug Reaction (RS-ADR) Signal Assessment: Development and Validation

**DOI:** 10.2196/35464

**Published:** 2022-10-06

**Authors:** Suehyun Lee, Jeong Hoon Lee, Grace Juyun Kim, Jong-Yeup Kim, Hyunah Shin, Inseok Ko, Seon Choe, Ju Han Kim

**Affiliations:** 1 Department of Biomedical Informatics College of Medicine Konyang University Daejeon Republic of Korea; 2 Seoul National University Biomedical Informatics (SNUBI) Division of Biomedical Informatics Seoul National University College of Medicine Seoul Republic of Korea; 3 Healthcare Data Science Center Konyang University Hospital Daejeon Republic of Korea

**Keywords:** adverse drug reaction, ADR, real-world data, RWD, real-world evidence, RWE, pharmacovigilance, PV, reference standard, pharmacology, drug reaction

## Abstract

**Background:**

Pharmacovigilance using real-world data (RWD), such as multicenter electronic health records (EHRs), yields massively parallel adverse drug reaction (ADR) signals. However, proper validation of computationally detected ADR signals is not possible due to the lack of a reference standard for positive and negative associations.

**Objective:**

This study aimed to develop a reference standard for ADR (RS-ADR) to streamline the systematic detection, assessment, and understanding of almost all drug-ADR associations suggested by RWD analyses.

**Methods:**

We integrated well-known reference sets for drug-ADR pairs, including Side Effect Resource, Observational Medical Outcomes Partnership, and EU-ADR. We created a pharmacovigilance dictionary using controlled vocabularies and systematically annotated EHR data. Drug-ADR associations computed from MetaLAB and MetaNurse analyses of multicenter EHRs and extracted from the Food and Drug Administration Adverse Event Reporting System were integrated as “empirically determined” positive and negative reference sets by means of cross-validation between institutions.

**Results:**

The RS-ADR consisted of 1344 drugs, 4485 ADRs, and 6,027,840 drug-ADR pairs with positive and negative consensus votes as pharmacovigilance reference sets. After the curation of the initial version of RS-ADR, novel ADR signals such as “famotidine–hepatic function abnormal” were detected and reasonably validated by RS-ADR. Although the validation of the entire reference standard is challenging, especially with this initial version, the reference standard will improve as more RWD participate in the consensus voting with advanced pharmacovigilance dictionaries and analytic algorithms. One can check if a drug-ADR pair has been reported by our web-based search interface for RS-ADRs.

**Conclusions:**

RS-ADRs enriched with the pharmacovigilance dictionary, ADR knowledge, and real-world evidence from EHRs may streamline the systematic detection, evaluation, and causality assessment of computationally detected ADR signals.

## Introduction

### Theories

An increasing number of studies have reported serious postmarket adverse drug reactions (ADRs) that were not discovered in Phase III clinical trials. Clinical trials are inherently limited in reflecting real-world settings where patients with diverse demographics and comorbidities take a variety of concurrent medications [1]. Real-world factors such as off-label medication prescriptions and irregular drug intake increase the risk of missing ADRs in clinical trials. Clinical trials have difficulty in identifying ADRs occurring in the real-world environment, such as delayed ADRs and effects from long-term drug exposure [2]. ADR-related medical costs for morbidity and mortality in the United States have been reported to be greater than US $75 billion per year [3,4]. Hence, the importance of postmarket drug-safety surveillance cannot be overemphasized. Drug-safety surveillance plays a role in managing and preventing potential ADRs and involves a wide range of activities that includes an entire cycle of collecting, analyzing, and monitoring related to ADRs. ADR signals exist in many forms, such as clinical signs, symptoms, diseases, or deaths. Spontaneous reporting systems, collecting suspected postmarket ADRs with causality assessments [5], are inherently biased.

### Prior Work

Computational methods for massively parallel detection of almost all drug-ADR interactions using real-world data (RWD), such as claims and multicenter electronic health records (EHRs), are emerging as relatively unbiased approaches [6-16]. However, validating massively detected ADR signals is challenging due to the lack of a “gold standard” or established reference set for all pairwise drug-ADR associations. In addition, determining a negative association is even more difficult than a positive one. Even the large, expert-curated reference standard provided by the major entities are disappointingly inadequate in correctly evaluating all computationally detected drug-ADR interactions. A reference standard involves a set of positive cases that are truly related to ADRs and negative controls that are highly unlikely to be associated. The reference standard should be formidable and have variety with multiple drugs and ADRs to ensure generalizability [17].

Coloma et al [10] developed a reference standard with 44 positive and 50 negative associations. The Observational Medical Outcomes Partnership (OMOP) presented a comprehensive compilation of 165 positive and 234 negative outcomes from their resources [18]. The EU-ADR presented 10 types of events associated with drug use, including 44 positive and 50 negative controls, based on a literature review [10]. Recently, Observational Health Data Sciences and Informatics published a knowledge base of 1000 drugs and 100 health outcomes of interest [19]. The Observational Health Data Sciences and Informatics group developed and tested the accuracy of an automated reference set to reduce manual curations [20]. Considering that previous studies [18-21] have relied mainly on literature and spontaneous reports, the coordination of evidence from different data sources is needed.

In silico ADR detection using RWD is much faster than reference standard development relying on expert curations. RWD analysis can potentially provide a reference standard for ADR signal evaluation. A systematic application of controlled vocabularies with rich semantics is essential for in silico pharmacovigilance (PV) using RWD. The controlled vocabulary–based ADR signal dictionary (CVAD) integrated controlled vocabularies with EHR data to improve PV [22]. The development of CVAD was motivated by previous research on massively parallel ADR signal detection algorithms using laboratory results and standard nursing statements, MetaLAB and MetaNurse [23]. Given the limited numbers of positive and negative reference sets, the correct validation of positive and negative drug-ADR associations among 101 precautionary drugs by thousands of ADR signals is challenging. A comprehensive reference standard is required for drug-ADR pairs, equipped with standard vocabulary annotations, in the emerging era of RWD and real-world evidence (RWE).

For prevention and management in PV, a strategy for integrating multiple data sources is preferred. Wei et al [24,25] combined RxNorm, Side Effect Resource (SIDER), MedlinePlus, and Wikipedia to compose a medication indication resource (MEDI). Gottesman et al [26] developed the Electronic Medical Records and Genomics network that advanced clinical informatics, genome science, and community consultation as a first step toward incorporating genomic information into routine health care delivery. Additionally, national-level projects are being carried out in several countries, or related research authorized, due to the need for a data-driven approach.

### Goal of This Study

A key challenge in drug-safety surveillance, regardless of data source, is that publicly available, reliable, and sufficiently large reference standards are needed. Although no definitive reference standard contains a complete set of ADRs, we intended to aggregate information from multiple data sources to constitute a set. In this study, we developed a reference standard for ADR (RS-ADR) for the comprehensive, efficient, and pragmatic evaluation of computationally detected massive ADR signals from RWD. RS-ADR integrates EHR term–related standard ADR terminologies, including those from the Medical Dictionary for Regulatory Activities (MedDRA) preferred terms (PTs), WHO Adverse Reactions Terminology (WHO-ART), Logical Observation Identifiers Names and Codes (LOINC), and International Classification of Diseases 10th Revision (ICD-10). We created the RS-ADR by aggregating massively parallel results of RWD and cross-validations for the positive and negative cases extracted from a multitude of health care organizations. Other PV resources, including OMOP and EU-ADR reference standards, the US Food and Drug Administration (FDA) Adverse Event Reporting System (FAERS) [27], and SIDER 4.1, were used as reference sets and augmented with controlled PV vocabularies to improve systematic causality assessments of drug-ADR associations. We tried to analyze and compare previously published reference sets, significantly increasing the number of cases, and developed RS-ADR by focusing on terminology standardization.

## Methods

### Development of Reference Sets for ADR Signal Evaluation

Given the lack of a “gold standard” to evaluate true and false ADR signals detected from PV studies, many researchers have attempted to compose ad hoc “gold standard” sets. Table 1 summarizes the different characteristics of the proposed “gold standard” sets by data source, number of drugs and ADRs, numbers of drug-ADR pairs, true positive and negative cases, controlled vocabularies, and evidence. The main objective of these studies in creating these reference standards was to evaluate the performance of the proposed algorithms. ADR signals were mainly defined by laboratory test results and clinical events, such as symptoms.

**Table 1:** Reference sets created and used by pharmacovigilance methodological studies.

Reference set	Data source	Drugs, n	ADRs^a^				Vocabulary
			ADRs, n	Drug-ADR pairs, n	Positive cases, n	Negative cases, n	
RS-ADR^b^	Laboratory test and event (symptom)	1344	4485	6,027,840	141,729	2330	ATC^c^, MedDRA^d^, WHO-ART^e^, LOINC^f^, and ICD^g^
Harpaz et al [13], 2012	Event (symptom)	44	38	137	62	75	RxNorm, and MedDRA
Yoon et al [14], 2012	Laboratory test	10	51	510	—^h^	—	None
Liu et al [15], 2013	Laboratory test	9	42	378	—	—	None
LePendu et al [16], 2013	Event (symptom)	78	12	193	28	165	MedDRA
Alvarez et al [28], 2010	Event (symptom)	267	—	—	532	—	MedDRA
Hochberg et al [29], 2009	Event (symptom)	35	—	—	6207	—	MedDRA
Ryan et al [30], 2012 (OMOP^i^ version 1)	Event (symptom)	10	9	90	9	44	None
Ryan et al [18], 2013 (OMOP version 2)	Event (symptom)	191	4	398	165	234	None
Coloma et al [10], 2013 (EU-ADR)	Event (symptom)	66	10	94	43	50	None
Boyce et al [19], 2014 (OHDSI^j^ knowledge base)	Event (symptom)	1000	100	100,000	—	—	ATC, RxNorm, and ICD

^a^ADR: adverse drug reaction.

^b^RS-ADR: reference standard for adverse drug reaction.

^c^ATC: Anatomical Therapeutic Chemical.

^d^MedDRA: Medical Dictionary for Regulatory Activities.

^e^WHO-ART: World Health Organization Adverse Reactions Terminology.

^f^LOINC: Logical Observation Identifiers Names and Codes.

^g^ICD: International Classification of Diseases.

^h^Not available.

^i^OMOP: Observational Medical Outcomes Partnership.

^j^OHDSI: Observational Health Data Sciences and Informatics.

The practical databases used in the study for constructing RS-ADRs were SIDER 4.1, OMOP, and EU-ADR. SIDER 4.1 contains the numbers of drugs, ADRs, drug-ADR pairs, and drug frequency entries from various references [31]. In addition, there are various databases (eg, Sentinel and the National Patient-Centered Clinical Research Network), but OMOP and EU-ADR are the most used in all fields of PV and provide an actual reference set. The researchers manually reviewed related references and finally selected the databases after being confirmed by clinicians. The OMOP database derived data from private contractors in the United States and EU-ADR derived data from European nationwide registries. Both were used for the identification of well-known drug associations and previously unknown signals [32].

**Figure 1:**
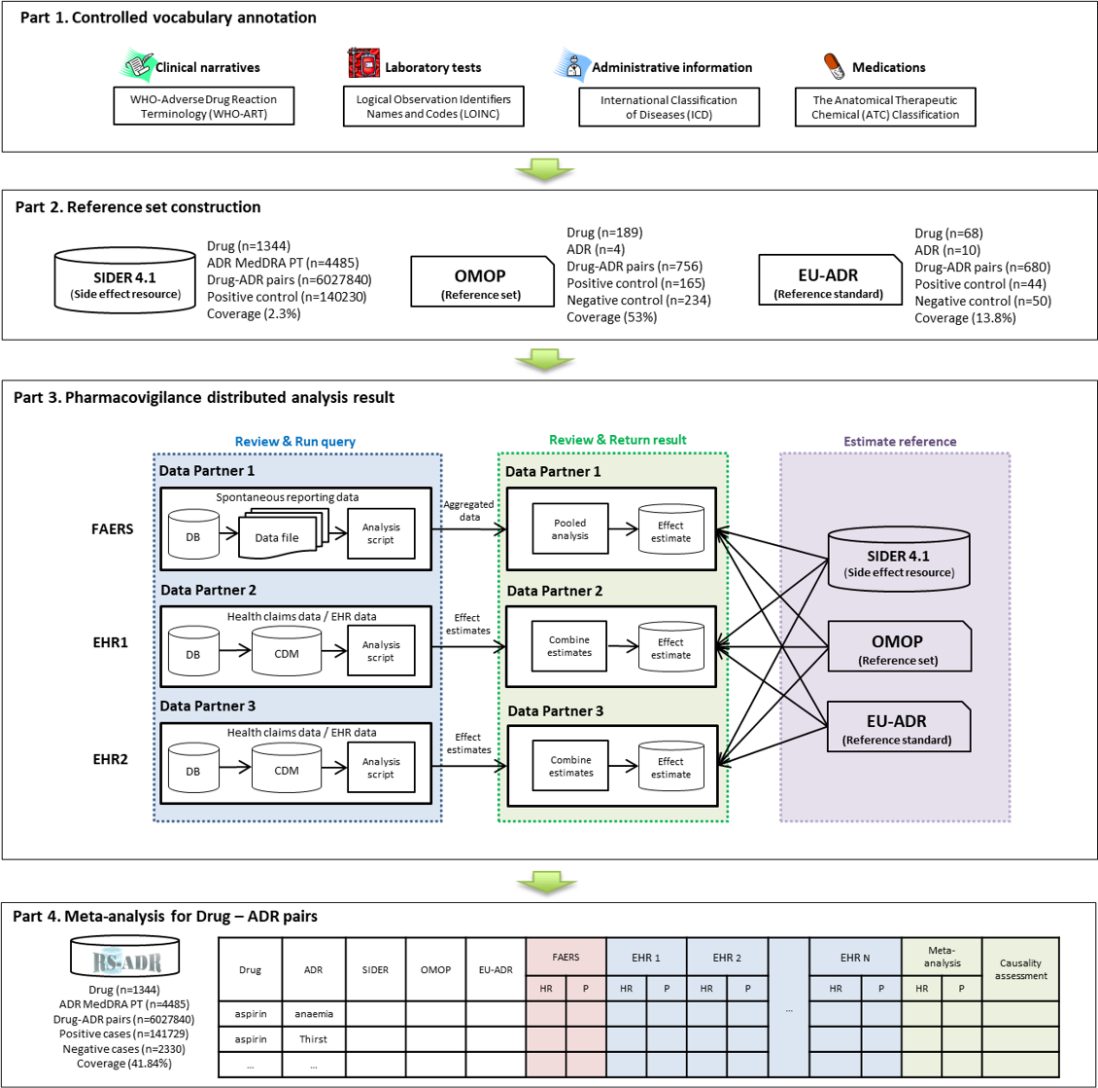
Flowchart of the construction of the RS-ADR, which uses electronic health record data (clinical narrative, laboratory tests, and disease classification). ADR: adverse drug reaction; CDM: common data model; DB: database; EHR: electronic health record; FAERS: Food and Drug Administration Adverse Event Reporting System; HR: hazard ratio; MedDRA: Medical Dictionary for Regulatory Activities; OMOP: Observational Medical Outcomes Partnership; PT: preferred term; RS-ADR: reference standard for adverse drug reaction.

A reference standard is essential for the evaluation of analysis results and systematic accumulation of evidence from comprehensive PV studies [13-31]. Therefore, we first created a reference standard based on the OMOP and EU-ADR projects. In all, 4 steps were used in the construction of the RS-ADR: (1) controlled vocabulary annotation, (2) reference set construction, (3) distributed analysis results, and (4) meta-analysis for drug-ADR pairs (Figure 1). The role of each part is elaborated in the following sections.

### Part 1: Controlled Vocabulary Annotation

A comprehensive annotation of controlled vocabularies that encompass disease classifications, laboratory tests, medications, and clinical narratives enables extensive EHR data exploration. Laboratory results have been used the most frequently for ADR signal detection in many studies. CVAD facilitates the use of a variety of data sources to detect ADR signals [22]. Clinical narratives such as International Council for Nursing Practice–based standard nursing statements (SNSs) of Seoul National University Hospital (SNUH) were mapped to WHO-ART, laboratory test results from SNUH or Ajou University Hospital were mapped to LOINC, administrative terms were mapped to ICD-10, and medications were mapped to ATC classifications. The mapping schemes involving narrative, laboratory, or administrative terms have been described in detail elsewhere [22].

### Part 2: Reference Set Construction

OMOP also provides a reference set, which is composed of 165 positive and 234 negative drug-ADR signal pairs, covering 53% of the 756 (189 × 4) pairs between 189 drugs and 4 ADRs [15]. The reference set of the EU-ADR project covers 68 drugs and 10 ADRs with 44 positive and 50 negative drug-ADR signal pairs, covering 13.8% [16]. The reference set prepared by SIDER 4.1 includes 140,230 positive pairs in MedDRA PTs for 1344 drugs and 4485 side effects without providing negative controls [31]. The coverage of SIDER 4.1 for drug-ADR pairs was 2.3%.

We mapped the 4 ADRs of OMOP and the 10 ADRs of EU-ADR to 4485 MedDRA terms in SIDER 4.1 using MedDRA synonyms (Unified Medical Language System Concept ID). We created a reference standard matrix for 1344 drugs and 4485 ADRs returning 6,027,840 drug-ADR pairs. The value of each cell of the reference matrix was filled with 0 for negative controls, 1 for positive controls, and 2 for unknowns. Negative controls were those known to not cause the outcomes, using case reports, case series, or observational evidence in OMOP and EU-ADR. Positive controls were extracted from the product labels in the US FDA “Black Box Warning” section in SIDER 4.1, OMOP, and EU-ADR (Figure 1).

### Part 3: Distributed PV Analysis Results

We benchmarked the analysis data from various institutions for PV [18] and integrated results from various resources such as spontaneous reports (ie, FAERS data), claims, and EHR data for developing RS-ADR. FAERS data from January 2012 to December 2018 were analyzed [27]. We performed the MetaNurse and MetaLAB analyses for the entire EHR data sets of 2 hospitals (SNUH and Konyang University Hospital) for SNSs and laboratory results using an advanced subject-sampling strategy for managing drugs, laboratory results, and SNSs. The detected ADR signals from the 2 EHR data sets were validated against SIDER 4.1 using 11,817 and 76,457 drug-ADR pairs, respectively [23]. We explored the relationship between drug-ADR pairs using spontaneous reports and EHR data. Table 2 shows the consensus template of our validation efforts for the “fluconazole-hypokalemia” association detected by the algorithms. Previous studies without annotated, controlled vocabularies experienced difficulty in evaluating their study results [33].

**Table 2:** Example of RS-ADR^a^ output for the association between “fluconazole” and “hypokalemia.”

Output, name			Example			
**Drug**						
		Drug		Fluconazole		
		ATC^b^ code		J02AC01		
**ADR^c^**						
		MedDRA^d^ PT^e^		Hypokalemia		
		System organ class		Metabolism and nutrition disorders		
**Part 1**						
	**Clinical narrative**					
		WHO-ART^f^		Hypokalemia		
		SNS^g^ terms at SNUH^h^		Serum potassium levels under normal | Hypokalemia		
		ICNP^i^		“mg/dL,” “not balanced,” and “fluid volume”		
	**Laboratory results**					
		LOINC^j^ ID		2823_3		
		LOINC common name		Potassium (moles/volume) in serum or plasma		
		SNUH laboratory test code		L3044		
		SNUH laboratory test name		Potassium (serum)		
		AJUH^k^ laboratory test code		35		
		AJUH laboratory test name		Potassium		
	**Disease classification**					
		ICD^l^ code		E87.6		
		ICD name		Hypokalemia		
**Part 2**						
	**Evidence source (0=negative control, 1=positive control, and 2=unknown)**					
		FDA^m^ product label**:** SIDER^n^		1		
		FDA product label and literature: OMOP^o^		2		
		FDA product label, literature, spontaneous data, and mechanism of action: EU-ADR		2		
**Part 3**						
	**Data partner: SNUH (EHR^p^-based MetaNurse)**					
		Hazard ratio		1.47		
		*P* value		<.001		
	**Data partner: SNUH (EHR-based MetaLAB)**					
		Odds ratio		3.04		
		*P* value		<.001		
	**Data partner: KYUH^q^ (EHR-based MetaLAB)**					
		Odds ratio		1.58		
		*P* value		<.001		
	**Data partner: FAERS^r^**					
		Reporting odds ratio		1.83		
		*P* value		<.001		
	**Data partner (N)**					
		Odds ratio		—^s^		
		*P* value		—		
**Part 4**						
	**Meta-analysis**					
		Odds ratio (95% CI)		1.69 (1.60-1.79)		
		Causality assessment		possible		

^a^RS-ADR: reference standard for adverse drug reaction.

^b^ATC: Anatomical Therapeutic Chemical.

^c^ADR: adverse drug reaction.

^d^MedDRA: Medical Dictionary for Regulatory Activities.

^e^PT: preferred term.

^f^WHO-ART: World Health Organization Adverse Reactions Terminology.

^g^SNS: standard nursing statement.

^h^SNUH: Seoul National University Hospital.

^i^ICNP: International Council for Nursing Practice.

^j^LOINC: Logical Observation Identifiers Names and Codes.

^k^AJUH: Ajou University Hospital.

^l^ICD: International Classification of Diseases.

^m^FDA: Food and Drug Administration.

^n^SIDER: Side Effect Resource.

^o^OMOP: Observational Medical Outcomes Partnership.

^p^EHR: electronic health record.

^q^KYUH: Konyang University Hospital.

^r^FAERS: Food and Drug Administration Adverse Event Reporting System.

^s^Not available.

### Part 4: Meta-analysis for Drug-ADR Pairs

We evaluated the drug-ADR pairs of the MetaLAB and MetaNurse analyses from multiple EHRs and compared with FAERS for causality assessments as follows: certain, probable/likely, possible, unlikely, or conditional/unclassified [10]. We applied a random-effects model for the meta-analysis of many results to manage the heterogeneous data characteristics of spontaneous reports and EHRs. To assess causality, we carried out expert reviews by having the experts refer to SIDER 4.1 and other existing references. Subsequently, PV-distributed analysis results generated by various health care organizations were collected for a causality assessment of each drug-ADR pair. With an increasing number of data partners providing study results, the causality assessment of each drug-ADR pair can be improved.

### Ethical Considerations

This study was approved by the Institutional Review Board of Konyang University Hospital (IRB no 2019-08-018).

## Results

### RS-ADR Statistics

The RS-ADR contained 1344 drugs and 4485 ADRs in terms of MedDRA PTs (Tables 3 and 4). The number of controlled vocabularies mapped to MedDRA PTs was for 1130 clinical narratives, 942 laboratory results, and 83 disease classifications. For positive controls, we found 140,230 drug-ADR pairs from SIDER 4.1, 1556 from OMOP, and 421 from the EU-ADR databases. The negative controls were 2801 and 349 drug-ADR pairs from OMOP and EU-ADR, respectively. ADRs were examined according to a variety of MedDRA system organ classes (SOCs) for clinical narratives, laboratory results, and disease classifications, covering 25, 23, and 16 of the 26 MedDRA SOCs, respectively (Multimedia Appendix 1). Although previous ADR studies predominantly analyzed laboratory results, we browsed 1762 integrative ADRs (ie, the intersection of clinical narrative, laboratory tests, and disease classification) with RS-ADR.

**Table 3:** RS-ADR^a^ statistics.

Statistic			Value, n
Drugs			1344
**ADRs^b^ (MedDRA^c^ preferred term)**			
	Total	4485	
	Clinical narrative	1130	
	Laboratory tests	942	
	Disease classification	83	
	Not mapped	2723	
Drug-ADR pairs (number of drugs × number of ADRs)			6,027,840

^a^RS-ADR: reference standard for adverse drug reaction.

^b^ADR: adverse drug reaction.

^c^MedDRA: Medical Dictionary for Regulatory Activities.

**Table 4:** RS-ADR^a^ statistics in comparison with other reference sets.

Statistic	SIDER^b^	OMOP^c^	EU-ADR
Positive controls, n	140,230	1556	421
Negative controls, n	—^d^	2801	349
Unknown drug-ADR^e^ pairs, n	5,887,610	6,023,483	6,027,070

^a^RS-ADR: reference standard for adverse drug reaction.

^b^SIDER: Side Effect Resource.

^c^OMOP: Observational Medical Outcomes Partnership.

^d^Not available.

^e^ADR: adverse drug reaction.

### An Example Application of RS-ADR

**Figure 2:**
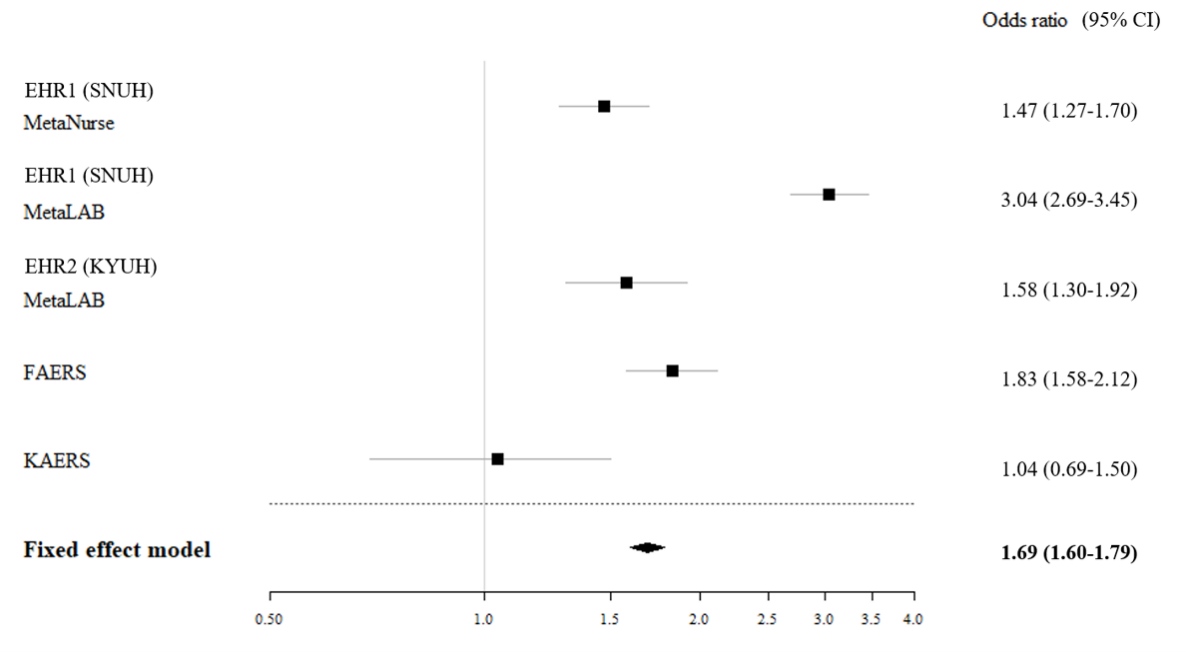
Example of the RS-ADR (part 3) for evaluating the association between the drug “fluconazole” and “hypokalemia” by using electronic health records (EHRs) from 2 hospitals (Seoul National University Hospital [SNUH] and Konyang University Hospital [KYUH]) and Food and Drug Administration Adverse Event Reporting System (FAERS) data. RS-ADR: reference standard for adverse drug reaction.

The process from part 1 to 4 for RS-ADR construction is briefly summarized as follows: first, the drugs and ADRs to be targeted; in part 1, term code confirmation; in part 2, the identification of contents described in the existing reference set; in part 3, analysis by data source; and in part 4, causality evaluation through meta-analysis. Table 2 shows a query result from RS-ADR for the association between “fluconazole” and “hypokalemia,” which explains the progress in stages from part 1 to part 4 in order. Part 1 consisted of 3 components: clinical narratives, laboratory results, and administrative data. Clinical narratives were annotated with WHO-ART “hypokalemia”; SNS “serum potassium levels less than normal”; and International Council for Nursing Practice “mg/dL,” “not balanced,” and “fluid volume” standard terms. Laboratory results were mapped to up to 6 tests, including LOINC “potassium (moles/volume) in serum or plasma.” The RS-ADR also indicated the direction of the test result to be higher or lower than the normal range. The administrative term mapped to ADR hypokalemia was the ICD-10 E87.6 code. Part 2 presented the evidence source of the drug-ADR association with positive controls, negative controls, and unknown evidence. Evidence sources could be FDA product labels, literature, spontaneous reports, and mechanisms of action. Part 3 designated the partner health care organizations where the ADR analysis data were collected. MetaLAB and MetaNurse analyses were included [23]. Finally, part 4 described how the causality between drug-ADR occurrence was assessed. A meta-analysis of the association between “fluconazole” and “hypokalemia” showed an odds ratio of 1.69 (95% CI, 1.60–1.79). In all, 2 EHRs and 2 spontaneous reporting data sets show the scalability and availability of the RS-ADR (Figure 2). The usability of RS-ADR can be enhanced by adding drug-ADR pairs using RWD analysis. The association between “fluconazole” and “hypokalemia” was assessed according to the WHO–Uppsala Monitoring Centre causality categories as “possible,” as this category included the criteria “event or laboratory test abnormality” (Table 2) [33].

### Improving Reference Standards Using RWE

Table 5 shows 4 drug-ADR pairs that were previously unknown in SIDER 4.1, OMOP, and EU-ADR. In this regard, we found that 2 of the drug-ADR pairs were added to Korean FDA ADR labels [34], which signals that they might have been determined as false positives. For example, famotidine was used in gastrointestinal conditions related to acid secretion (eg, gastric ulcers) and gastroesophageal reflux disease [35]. The novel “famotidine–hepatic function abnormal” pair discovered by RS-ADR was successfully validated by 2 institutional EHRs and by US FAERS [35]. Moreover, according to the Micromedex [36] database and a study by Gupta et al [37], we found that the famotidine–hepatic function abnormal pair had been documented as a possible ADR. The RWD/RWE perspective suggests that the novel finding may indeed indicate a true positive supported by multi-institutional cross-validations. We performed the same analysis for clozapine and diclofenac and found reasonable support (with reservations) for the potential drug-ADR pairs “clozapine–hepatic function abnormal,” “diclofenac-angioedema,” and “diclofenac–face edema” (Table 5).

**Table 5:** RS-ADR^a^ evidence of how significant the drug-ADR^b^ pairs are using the EHR^c^ data of 2 hospitals (Seoul National University Hospital [SNUH] and Konyang University Hospital [KYUH]) and Food and Drug Administration Adverse Event Reporting System (FAERS) data.

Drug	ADR	RS-ADR									Reference
		SNUH				KYUH		FAERS			
		EHR-based MetaNurse		EHR-based MetaLAB		EHR-based MetaLAB					
		HR^d^	*P* value	OR^e^	*P* value	OR	*P* value	OR	*P* value		
Famotidine	Hepatic function abnormal	1.79	<.001	2.19	.003	1.11	.008	3.97	<.001	Gupta et al [37], 2009	
Clozapine	Hepatic function abnormal	0.55	.04	1.38	.01	—^f^	—	1.02	.85	Wu Chou et al [38], 2014	
Diclofenac	Angioedema	0.96	.47	—	—	—	—	5.13	<.001	Pise and Padwal [39], 2015	
Diclofenac	Face edema	2.38	.20	—	—	—	—	1.95	<.001	Jha et al [40], 2015	

^a^RS-ADR: reference standard for adverse drug reaction.

^b^ADR: adverse drug reaction.

^c^EHR: electronic health record.

^d^HR: hazard ratio.

^e^OD: odds ratio.

^f^Not available.

### Web-Based RS-ADR Explorer

**Figure 3:**
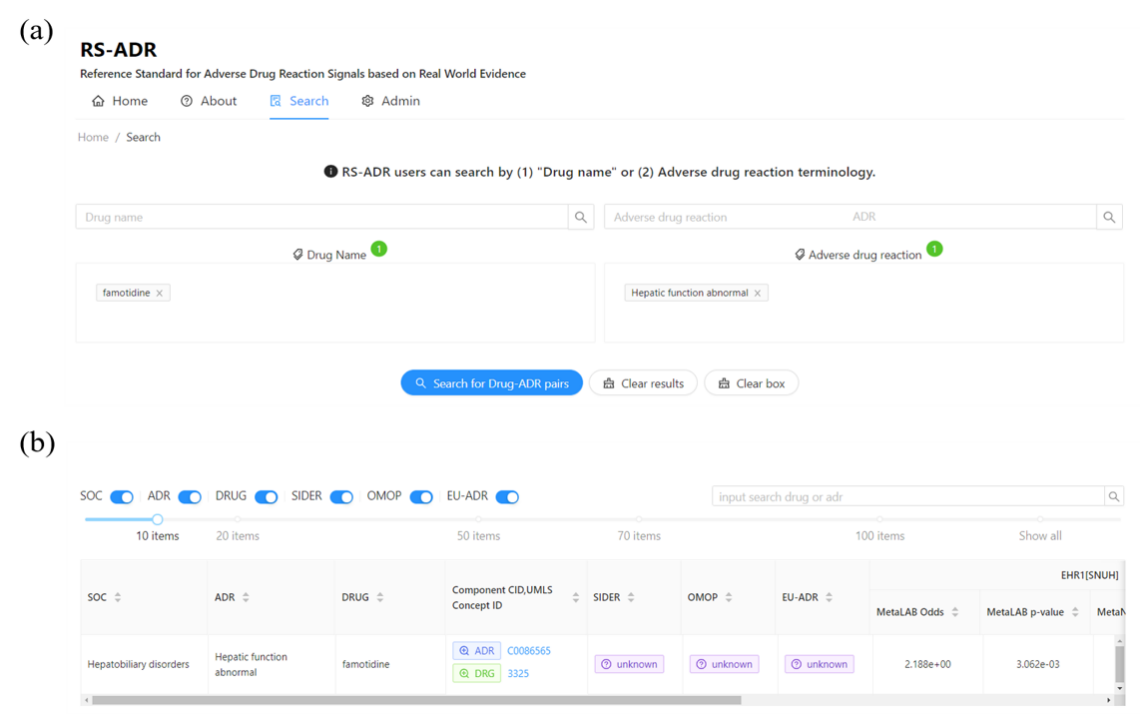
User interface for the RS-ADR for exploring the drug-ADR relationship. (A) Drug-ADR search; (B) Example of RS-ADR query: association between “famotidine” and “hepatic function abnormal.” ADR: adverse drug reaction; CID: component identification; OMOP: Observational Medical Outcomes Partnership; RS-ADR: reference standard for adverse drug reaction; SIDER: Side Effect Resource; SOC: system organ class; UMLS: Unified Medical Language System.

To provide a semantically enriched ADR dictionary for postmarket drug safety research and enable multicenter EHR-based extensive ADR signal evaluation, we developed a web-based search interface for RS-ADR to explore drug-ADR associations [41] (Figure 3). Figure 3 shows the drug-ADR search functions and the results of a “famotidine–hepatic function abnormal” query. Users can search for interesting drug-ADR pairs in combination; each search function adds similar words using drop-down menus. A button clears the drug-ADR combinations and results to facilitate searching. Search results appear in the order of SOC, ADR, drug, additional information (component identification for drug and Unified Medical Language System concept ID for ADR), comparison of reference standards (SIDER, OMOP, and EU-ADR), and each result of the EHR and FAERS (odds ratio and *P* value). Parts 3 and 4 of the RS-ADR have a structure that allows researchers to add and update their results to improve the RS-ADR.

## Discussion

### Principal Findings

In this study, we demonstrated the possibility of creating an RWD-based RS-ADR. We integrated various standard vocabularies to facilitate the use of different institutional EHR databases along with other PV resources, such as SIDER 4.1, OMOP, and EU-ADR. Integrative analysis of heterogeneous real-world clinical information requires a standard vocabulary to correctly interpret study results.

The reference sets of OMOP and EU-ADR [15,16] are difficult to apply directly in PV research, because they only provide information about the relationships between the selected drugs and ADRs. To use these reference sets, each observational database should be reconstructed and annotated using controlled vocabularies by the researchers. The RS-ADR approach facilitates the accumulation of RWD-driven evidence extracted from various sources, including many EHRs and claims databases. The scope of detectable ADRs was widely expanded by RS-ADR using FDA structured product labels and low ADR concept levels (eg, MedDRA PTs). A low ADR concept level is most commonly used in the standard terminology system to explain detailed symptoms such as MedDRA PTs. RS-ADR complements this limitation by establishing a reference standard using 1344 drugs and 4485 ADRs. The RS-ADR approach used in this study is not as biased toward positive findings as other PV resources but is balanced between positive and negative drug-ADR associations due to its unbiased computational approach. Multimedia Appendix 1 shows the distribution of MedDRA PT–annotated ADRs detected using clinical narratives, laboratory results, and administrative terms grouped by SOCs. The SOCs “infections and infestations,” “psychiatric disorders,” and “eye disorders” exhibit many ADRs that are difficult to detect from laboratory results only and require clinical narratives, nursing statements, and administrative terms in the RS-ADR. The ADRs in “musculoskeletal and connective tissue disorders” and “ear and labyrinth disorders” SOCs could only be found using clinical narratives.

### Limitations

Our study has some limitations. SIDER 4.1 provides inadequate information about postmarket ADRs as it comprises public documents and package inserts. The 4 ADRs of the OMOP and 10 ADRs of the EU-ADR project may emphasize ADRs of more frequently or chronically used drugs, which are also clinically important. The use of integrative ADR references such as SIDER 4.1, OMOP, and EU-ADR in the RS-ADR complements the limitations of each resource. Although the RS-ADR went through interevaluator agreement, expert evaluation was substantially limited, and continuous review and updates are required. When integrated with multicenter and multinational data, RS-ADR becomes a meaningful RWE-based reference standard for evaluating ADR signals. Underlying the use of a reference standard for method evaluation is the assumption that negative controls are exchangeable with positive controls [10,18]. Adding drug-ADR pairs from various studies to the RS-ADR can increase its evidence base and is a topic of future research. In addition, considering the continuous RS-ADR update, it is planned to manage the analysis of new drugs and whether to discontinue the use of existing drugs. For national use, since the Korean Ministry of Food and Drug Safety is conducting related research (eg, multicenter analysis using common data model–based EHR, analyzing each drug-ADR pair), our team will contemplate various utilizations of RS-ADR for collecting and evaluating the research. Conversely, recent attempts to study ADRs related to herbal medicines have steadily increased [42-44], and we consider that it may be possible to apply RS-ADR construction to the field of herbal medicine in the future.

### Conclusions

RS-ADR enriched with the PV dictionary, knowledge, and RWE can streamline the systematic detection, evaluation, and causality assessments of computationally detected ADR signals. Through RS-ADR, evidence related to ADRs can be prepared as much as possible before the clinical evaluation stage, and we could identify more cases based on actual medical center data—RWD. In addition, since we considered the standardization of terms for drugs and ADRs, it is highly useful when adding medical center or other resources in the future. It is applicable not only to ADR studies but also to a variety of health outcomes and health care database utilization studies.

## Appendix

Multimedia Appendix 1 Number of adverse drug reactions in MedDRA preferred terms of the RS-ADR for each system organ class. ADR: adverse drug reaction; MedDRA: Medical Dictionary for Regulatory Activities; RS-ADR: reference standard for adverse drug reaction.

## Abbreviations

ADR: adverse drug reaction
CVAD: controlled vocabulary–based ADR signal dictionary
EHR: electronic health record
FAERS: Food and Drug Administration Adverse Event Reporting System
FDA: Food and Drug Administration
ICD-10: International Classification of Diseases 10th Revision
LOINC: Logical Observation Identifiers Names and Codes
MedDRA: Medical Dictionary for Regulatory Activities
OMOP: Observational Medical Outcomes Partnership
PT: preferred term
PV: pharmacovigilance
RS-ADR: reference standard for adverse drug reaction
RWD: real-world data
RWE: real-world evidence
SIDER: Side Effect Resource
SNS: standard nursing statement
SNUH: Seoul National University Hospital
SOC: system organ class
WHO: World Health Organization
WHO-ART: World Health Organization Adverse Reactions Terminology
