# Supplementary figures and images for: A Data-Driven Reference Standard for Adverse Drug Reaction (RS-ADR) Signal Assessment: Development and Validation
Source: J Med Internet Res. 2022 Oct 6;24(10):e35464. doi: 10.2196/35464 (PMC9585444; doi:10.2196/35464)

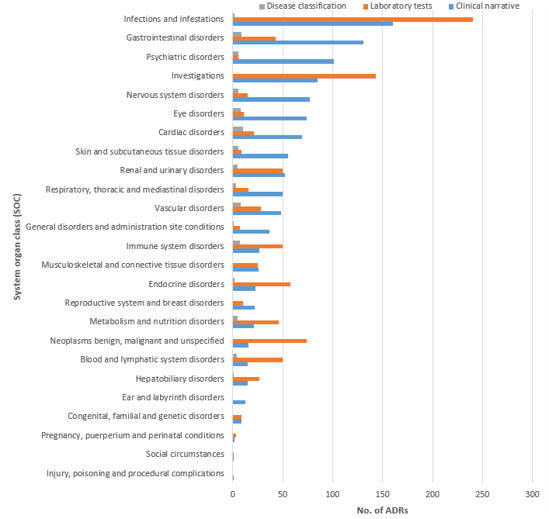

Supplement: Multimedia Appendix 1 [file jmir_v24i10e35464_app1.png]
